# The impact of endocrine disrupting chemicals diethylstilbestrol and ketoconazole on lipid storage in in vitro matured bovine cumulus-oocyte complexes^†^

**DOI:** 10.1093/biolre/ioag119

**Published:** 2026-06-05

**Authors:** Konstantina Asimaki, Chris H A Lest van de, Richard Wubbolts, Jeroen W A Jansen, J Bernd Helms, Hilde Aardema, Majorie B M Duursen van, Barend M Gadella

**Affiliations:** Department Environmental Health and Toxicology, Amsterdam Institute for Life and Environment, Vrije Universiteit Amsterdam, Amsterdam, Netherlands; Department of Population Health Sciences, Faculty of Veterinary Medicine, Utrecht University, Utrecht, Netherlands; Department of Biomedical Health Sciences, Faculty of Veterinary Medicine, Utrecht University, Utrecht, Netherlands; Department of Biomedical Health Sciences, Faculty of Veterinary Medicine, Utrecht University, Utrecht, Netherlands; Department of Biomedical Health Sciences, Faculty of Veterinary Medicine, Utrecht University, Utrecht, Netherlands; Department of Biomedical Health Sciences, Faculty of Veterinary Medicine, Utrecht University, Utrecht, Netherlands; Department of Population Health Sciences, Faculty of Veterinary Medicine, Utrecht University, Utrecht, Netherlands; Department Environmental Health and Toxicology, Amsterdam Institute for Life and Environment, Vrije Universiteit Amsterdam, Amsterdam, Netherlands; Department of Population Health Sciences, Faculty of Veterinary Medicine, Utrecht University, Utrecht, Netherlands; Department of Biomedical Health Sciences, Faculty of Veterinary Medicine, Utrecht University, Utrecht, Netherlands

**Keywords:** endocrine disrupting chemicals, diethylstilbestrol, ketoconazole, bovine, cumulus-oocyte complex, in vitro, lipid droplets, lipidomics, cholesterol, steroidogenesis

## Abstract

Endocrine disrupting chemicals (EDCs) impact female fertility, and their widespread presence demands deeper understanding of their effects on reproduction. In vitro maturation of bovine cumulus-oocyte complexes (COCs) serves as a relevant reproductive toxicology model aligned with the 3R principle. Given the link between lipid metabolism and COC quality, the possibility that lipid droplets (LDs) in cumulus cells (CCs) and overall COC lipid content are susceptible to EDC disruption was tested. COCs were exposed to diethylstilbestrol (DES; estrogen receptor agonist) and ketoconazole (KTZ; steroidogenesis inhibitor), during in vitro maturation. Neutral lipid dyes were used for LD visualization (count, size, area, and clustering) in CCs. Lipidomic analysis using liquid chromatography–mass spectrometry was conducted separately on oocytes and CCs. Of all tested DES concentrations (i.e., 10^−9^ M, 10^−7^ M, 10^−5^ M), 10^−5^ M DES reduced the total LD area per CC, associated with decreased LD count and increased size. KTZ at all tested concentrations (i.e., 10^−8^ M, 10^−7^ M, 10^−6^ M) reduced LD size and enhanced clustering. Lipidomic analysis revealed the selective depletion of cholesteryl esters in CCs exposed to 10^−5^ M DES or 10^−6^ M KTZ, with minimal changes in other neutral lipid classes. Thus EDCs can disrupt lipid storage and LD dynamics in bovine COCs. We discuss these new findings with our previous work: (i) DES-induced steroidogenesis while (ii) in contrast KTZ blocked steroidogenesis in CCs. Together, the data highlight the COC lipid metabolism as a novel EDC target, warranting further study due to potential implications for female fertility.

## Graphical Abstract

**Graphical Abstract:**
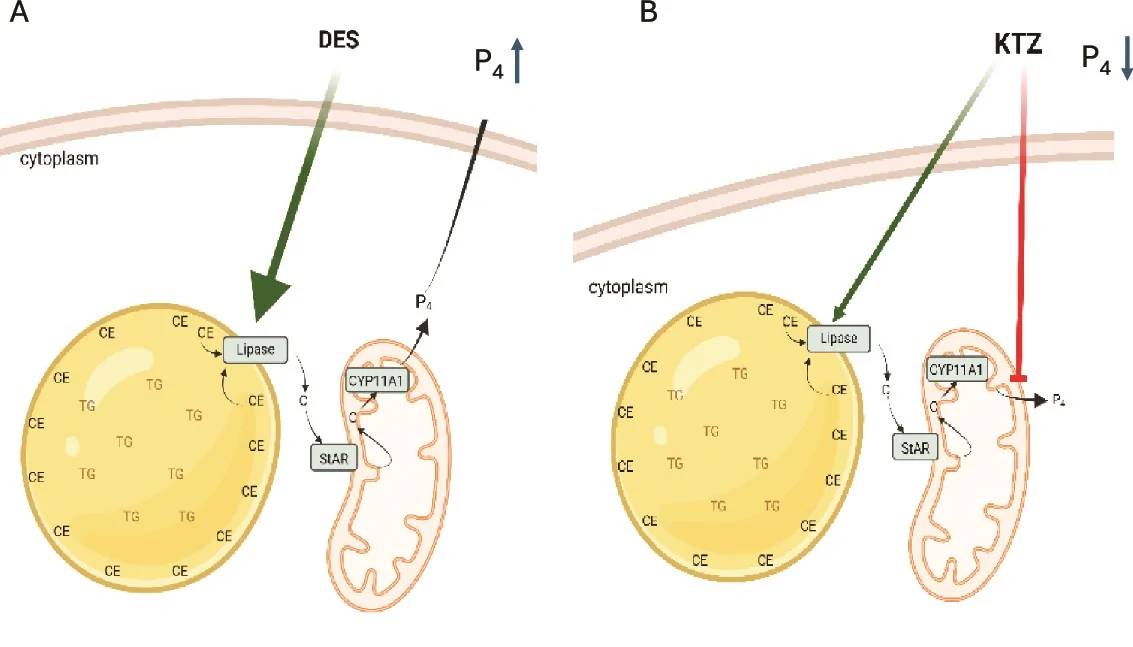

## Teaser

Diethylstilbestrol and ketoconazole disrupt lipid storage and lipid droplet dynamics in bovine COCs in vitro, highlighting the need to clarify EDC impacts on COC lipid metabolism and potential fertility consequences.

## Introduction

Humans and animals are exposed to chemicals through various sources, ranging from daily essentials such as cleaning and personal care products, to polluted air, water, and soil. Some of these chemicals are endocrine disrupting chemicals (EDCs). According to the world health organization (WHO), an EDC is “an exogenous substance or mixture that alters function(s) of the endocrine system and consequently causes adverse health effects in an intact organism, or its progeny, or (sub)populations” [1]. Since female reproduction depends on the endocrine system, it may be impaired by EDC action. In fact, in vitro and animal studies show that different aspects of female reproduction such as follicular count, hormone production, and ovulation, can be disrupted by EDCs [2, 3]. In the human population, a direct causal link between EDC exposure and adverse reproductive outcomes is more difficult to establish, though supporting evidence is increasing [4]. Notably, epidemiological studies have correlated the presence of EDCs in biological fluids (e.g., blood, urine, serum, and follicular fluid) with adverse reproductive outcomes (e.g., infertility, preterm delivery, and spontaneous abortion) in in vitro fertilization (IVF) patients, as well as other reproductive disorders like endometriosis and polycystic ovary syndrome [2, 5–10]. To protect female reproductive health, regulatory chemical safety assessment employs various in vivo and in vitro assays to identify EDCs causing reproductive toxicity. However, the currently used assays do not pinpoint effects specifically on the oocyte and its surrounding cumulus cells (CCs). The need for their inclusion in toxicological testing strategies is supported by the integral role of the cumulus-oocyte complex (COC) in female reproduction and by the steroidogenic capacity of CCs that makes them probable EDC targets [11].

Steroidogenesis is dependent on the supply of cholesterol, the precursor of all steroid hormones. There are three routes for cells to obtain cholesterol; uptake via a receptor-mediated endocytosis of lipoproteins, de novo synthesis, or hydrolysis of cholesteryl esters (CE) that are stored in lipid droplets (LDs). Structurally, LDs consist of a neutral lipid core, mainly of triacylglycerols (TG), and CE, enclosed by a monolayer of phospholipids and cholesterol. The neutral lipid composition of LDs is variable under diverse conditions and between cell types [12] and the periphery of LDs is decorated with LD-specific proteins [13, 14]. LDs may remain tethered to the endoplasmatic reticulum (ER) or be transported within the cytoplasm, and can interact and connect via different specific contact sites to multiple cell organelle membranes (e.g., lysosomes, peroxisomes, mitochondria) [15].

Various roles have been attributed to LDs in cells, including lipid storage as an energy source, participation in lipid homeostasis and cell signaling, and being a source for membrane components. In the COC, LDs present in CCs have an additional role in protecting the enclosed oocyte from lipotoxicity. For instance, excessive levels of specific saturated free fatty acids have been related to cellular stress in CCs and reduced oocyte competence [16, 17]. CCs can protect the oocyte against those potentially toxic free fatty acids via the stearoyl-Coa desaturase enzyme that catalyzes the formation of unsaturated oleic acid, resulting in storage of fatty acids in LDs and the maintenance of oocyte developmental competence [18–20]. Interestingly, LDs in CCs also contribute to steroidogenesis, when their endogenous CEs are mobilized via the activation of a cholesteryl-ester hydrolase, an enzyme present on the lipid monolayer surface of LDs [21]. The liberated cholesterol can become available for the steroidogenic enzymes present within mitochondria, via LD-mitochondrial membrane contact sites. Multiple proteins have been implicated in cholesterol transport within mitochondria, including steroidogenic acute regulatory protein (StAR) and translocator protein (TSPO). These proteins, localized at the outer mitochondrial membrane, can bind cholesterol through specialized domains and transport it from the outer to the inner mitochondrial membrane [22]. There, cholesterol side-chain cleavage enzyme (P450scc or CYP11A1) converts imported cholesterol to pregnenolone, which can be further metabolized into other steroid hormones [23].

A link between LDs and steroidogenesis has been demonstrated in both rodent testis and bovine ovaries [24, 25]. Cholesterol mobilized from LDs can be imported into mitochondria and subsequently become converted to pregnenolone StAR and CYP11A1, respectively. There is growing evidence that, both in the testis and the ovary, endogenously stored cholesterol is the main precursor for steroidogenesis [24–27].

Considering the various roles that LDs serve in CCs and their significance to COC function, it is important to discern how EDCs may affect LD dynamics. Thus far, EDC effects on neutral lipid content or lipid droplet formation and organization in CCs or oocytes have not been studied well. However, EDCs have been investigated in the context of metabolic diseases and have been implicated in lipid homeostasis dysregulation [28]. Specifically, various EDCs have been shown to alter lipid accumulation and lipid droplet formation in adipocytes and hepatocytes [29–31]. It is therefore plausible to hypothesize that EDCs may also affect lipid metabolism in COCs.

Previously, we have tested a bovine in vitro oocyte maturation model for screening and identifying EDCs for their potential toxic effects on the maturing COC [32, 33]. The bovine species was selected because of its strong similarity with humans in terms of oocyte maturation and early embryo development [34, 35]. Moreover, the herein used slaughterhouse material (i.e., cow ovaries) for COC collection supports the 3Rs principle to refine, reduce, and replace experimental animals. In the current study, two well-known EDCs having distinct mechanisms of action, diethylstilbestrol (DES; an estrogen receptor agonist) and ketoconazole (KTZ; steroidogenesis inhibitor), were selected as testing examples for potent EDCs with well-described endocrine action [36]. We showed in the past that DES or KTZ exposure of in vitro maturing bovine COCs only resulted in marginal effects on classical oocyte quality parameters, such as oocyte nuclear maturation and developmental competence, oocyte and CC apoptosis/necrosis, CC layer expansion, and steroidogenesis [32, 33]. Interestingly, both EDCs had an impact on steroidogenesis in cumulus cells, with DES increasing pregnenolone and progesterone production [32], while KTZ abolished the production of these hormones [32].

We now—as a follow-up of these two studies [32, 33]—report via studies performed with the same bovine in vitro maturation model that DES and KTZ elicited effects on neutral lipid storage and lipid mobilization in and from lipid droplets during in vitro maturation of COCs. The results are discussed in the light of previously reported effects of DES [32] and KTZ [33] on aspects of COC maturation and their effects on steroidogenesis.

## Methods

### Oocyte maturation and EDC exposure

COC isolation and culture was carried out as previously described in [32]. In short: ovaries collected from slaughtered cows in a local slaughterhouse were transported immediately to the lab in a thermal insulated box. The fluid of follicles (2–8 mm diameter) was aspirated and COCs with a healthy cumulus investment and a minimum of 3 cumulus cell layers, without signs of atresia, were recovered. COC maturation was performed for 24 h (5% CO_2_, 38°C) in NaHCO_3_-buffered maturation media M199 (#31100-027; GIBCO, Grand Island, NY, United States) supplemented with 0.05 IU/mL rec hFSH (Organon, Jersey City, NJ, United States), 0.1 μM cysteamine M9768 (Sigma-Aldrich, St. Louis, MO, United States), 10 ng/mL EGF E4127 (Sigma-Aldrich), and 10 IU/ml penicillin–streptomycin (GIBCO). COCs were exposed to DES (D4628-1G; Sigma-Aldrich; 10^−9^ M, 10^−7^ M or 10^−5^ M) or KTZ (K1003; Sigma-Aldrich; 10^−8^ M, 10^−7^ M or 10^−6^ M) during the entire maturation period of 24 h. Vehicle controls were 0.01% v/v dimethylsulfoxide (DMSO) and 0.1% v/v DMSO, for DES and KTZ, respectively. The amount of COCs and their respective total of cumulus cells used per experiment are available in Supplementary Table S1.

### Neutral lipid dye staining

Mature COCs were washed in phosphate-buffered saline (PBS), then fixed in 4% paraformaldehyde (PFA) and washed for 15 min with PBS. Staining was performed at room temperature. Specifically for the dyes BODIPY 493/503 and Nile Red, a permeabilization step (0.2% v/v Triton-X in PBS, 30 min) was carried out prior to staining while the permeabilization was omitted for LD540 staining. COCs were stained with either 0.1 μg/mL LD540 (30 min; gift from Prof. Christoph Thiele, LIMES-Institute of the University of Bonn, Germany) [37] or 20 μg/ml BODIPY 493/503 (1 h, Molecular Probes Europe, Leiden, the Netherlands), or 10 μg/mL Nile Red (2 h; Sigma Aldrich). Stained COCs were then washed 3 times for 3 min in 0.3% w/v PVP/PBS. Nuclear staining for all COCs was carried out with 20 μg/mL Hoechst 33342 (B2261; Sigma-Aldrich) for 45 min and then COCs were washed once in PBS. Stained COCs were then mounted with antifading fluid Vectashield (Vector Laboratories, Newark, CA, United States) on microscope slides carrying petroleum jelly bridges (formed by applying petroleum jelly onto the glass slide using a syringe) that serve to create spacing with the coverslip. All imaged COCs were as a routine checked for the incidence of nuclear fragmentation (a sign of apoptosis, visualized by Hoechst 33342 staining). None of the KTZ or DES conditions induced nuclear fragmentation as was reported before [32, 33].

### Confocal imaging and image analysis

COC images of 1024 × 1024 were collected with a Nikon A1R confocal scanning microscope (Nikon, NY, United States) using a quad band dichroic filter (405 nm, 488 nm, 561 nm, and 647 nm). Nuclei images were collected on the Hoechst 33342 channels with the 405 nm excitation wavelength laser (emission filter 450/50 nm) and neutral lipid dyes with the 488 nm excitation wavelength laser (emission max: BODIPY 493/503 at 503 nm—collected with a 515/30 nm emission filter, Nile Red at 580 nm and LD540 at 560 nm—both detected with a 595/50 emission filter). Image channels were recorded in a sequential illumination mode using a 100× oil immersion magnification lens (NA = 1.45). Three different regions of the cumulus cell investment of each COC were imaged. The first region was adjacent to the corona radiata, the second in the middle of the cumulus cell layer, and the third towards the outer cumulus cell layer. For each region, Z stack imaging was performed top-to-bottom (approximately 15 μm) with a 1 μm step-size.

**Figure 1:**
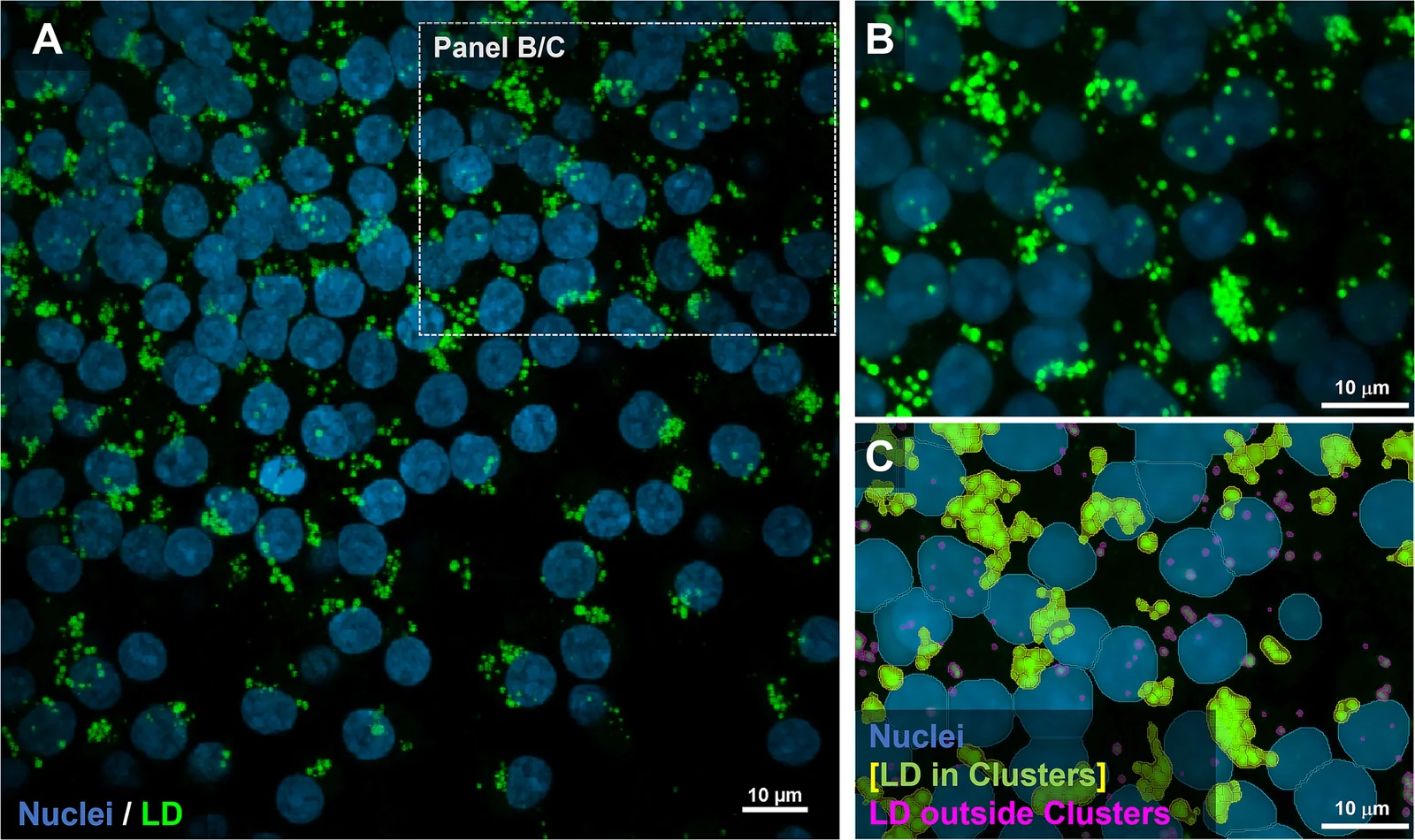
Representative image showcasing the analysis of cumulus cell images stained with LD540 for LD parameter characterization. (A) Overview with large oval nuclei that were Hoechst 3342 stained, and small ball shaped lipid droplets (LD) that were LD540 stained in. (B) Shows zoomed box in panel A as inset. (C) Representative segmentation result of the script for nuclei, LD classes defined as clusters by merged regions after an extra Gaussian filtering with a sigma of 3. The resulting clusters are outlined, their Watershedded enclosed LD units are depicted in overlays and outlined, the LD class located outside the clusters are outlined.

An automated method for image analysis was developed in NIS Elements software using the GA3 module (version 5.1, Nikon, NY, United States), adapted separately for LD540 and BODIPY 493/503, and Nile Red. Two object sets were identified, namely the nucleus and the LD. LDs were further analyzed and divided in two distinct subpopulations of (a) clustered LDs and (b) dispersed LDs (see Figure 1, illustrating the three different identified object classes). Output images were generated to visualize the segmentation process (for a representative output image, see Figure 1B). A brief overview of the image processing is given here and the detailed script as a general analysis 3 (.ga3) file in Supplementary file 2 (LD540, BODIPY 493/503) and Supplementary file 3 (Nile Red). The relevant HTML files can also be found in Supplementary file 4 and Supplementary file 5, respectively.

#### Nucleus detection

Segmentation was optimized to isolate nuclei using the Hoechst channel to allow normalization for the number of cells from a maximal intensity projected representation of the imaged volume. A preprocessing step for denoising and background subtraction was carried out by median filtering with a filter size of 7 pixels and using the rolling ball algorithm (9 μm radius). Maximum intensity projections were then processed by unsharp masking (power = 0.8, area = 1.7). Segmentation was performed by bright spot detection (2 μm, contrast 0.001, intensity 10) and further object area filtering was applied (from 5 to 219.6 μm^2^). Count of nuclei in the field of view was the primary output of this analysis. For an example of nucleus identified object detection, see Figure 1B.

#### LD parameter detection in cumulus cells using fluorescent neutral lipid dyes

Four LD parameters were assessed in bovine CCs. These parameters were calculated by analyzing confocal images of CCs stained with neutral lipid dyes and include LD count (per CC), LD clustering (% of total LDs), LD size (μm^2^), and total LD area per CC (μm^2^).

Background subtraction was carried out by Median filtering with a filter size of 2 pixels before using the Rolling Ball algorithm (2.51 μm radius). This corrected image (LD1) was duplicated, and a Regional Maxima filter (circular kernel of 3 pixels) was applied before Maximal Intensity Projection. Next, segmentation by bright spot detection (0.25 μm, contrast 0.1, intensity 300), and further object size filtering was applied (>25 pixels). Count and size of all lipid droplets within the field of view were the main output of this analysis. LD count per cumulus cell was calculated as the ratio of all lipid droplets counted within an image, to the total nuclei count of that image.

#### LD cluster detection

For lipid droplet cluster detection, the corrected LD1 image stack was subjected to Maximal Intensity Projection and a Gaussian blurring filter (σ = 3). Segmentation was performed by bright spot detection (2 μm, contrast 0.1, intensity 250–10 000) and binary circular closing (0.124 μm radius) to merge adjacent objects, as a means to define clusters by size filtering (between 4 and 5000 pixels) in the projected images. This analysis provided the counts of LDs within the field of view that resided in the clustered masks and allowed calculation of clustered vs. dispersed LDs. Clustered LDs (see representative Figure 1D) refer to the class of LDs within a cluster, and dispersed LDs (see representative Figure 1E) refer to the class of LDs that are dispersed within the cell cytoplasm without participating in a cluster. LD clustering was calculated as the percentage of LDs participating in a cluster to the total LD count within the field of view. LD area was calculated and is reported for all LDs, but also separately for clustered and dispersed LDs. An additional calculation of the total LD area per cumulus cell (*LD count* x *mean LD size*) and intensity measures in Summed Intensity Projections of the stacks were also carried out by the script.

### Lipid extraction from cumulus cells and oocytes

Lipids were extracted using the original Bligh and Dyer extraction method [38] that was adapted for small volumes using an internal glass insert having a total volume of 250 μL, as follows:

Cumulus cells were isolated from 10 COCs and transferred to a glass insert in a volume of 125 μL (in PBS buffer with 0.1% fatty acid free BSA). This volume was mixed with 100 μL CHCl_3_:methanol 1:2 (v/v) containing 10 picomole of the reference lipid sitosterol, and 10 picomole of the reference lipid 15:0-15:0-15:0-triacylglycerol. The lipids were extracted for 20 min by repeated pipetting at 0, 10, and 20 min. Centrifugation was carried out for 5 min at 2000× *g*. The supernatant containing the full lipid extract was transferred to a clean glass insert. The remaining glass insert with the protein precipitate was discarded, and 50 μL MilliQ-water and 50 μL CHCl_3_ were added and mixed by pipetting at 0, 10, and 20 min. A second centrifugation was performed, and two phases were separated. The lower organic phase contained primarily chloroform and ±90% lipids, while the upper aqueous phase contained the remaining ±10% of lipids. The lower phase was transferred to a clean glass insert. Lipids in the remaining upper phase were extracted by addition of 50 μL CHCl_3_ and repeated pipetting at 0, 10, and 20 min. After centrifugation for 5 min at 2000× *g*, the new lower organic phase was transferred to the previously isolated organic phase. The extracts were evaporated to dryness under streaming of N_2_ gas.

### LC–MS analysis of lipid extracts

Before liquid chromatography–mass spectrometry (LC–MS) analysis, the dried lipid extracts were resolubilized in 100 μL CHCl_3_:methanol 1:1 (v/v). LC–MS analysis was performed on a Q-Exactive HF mass spectrometer (Thermo Scientific, Waltham, MA, United States) coupled to a Vanquish Dual autosampler, column oven and pump (Thermo Scientific); 10 μL aliquots were injected directly onto a Kinetex C8 column (2.6 μm, 150 × 3.00 mm; Phenomenex, Torrance, CA, United States), column temperature was maintained at 40°C. Gradient elution was performed from methanol/water 1:1 (v/v) to methanol/isopropanol 4:1 (v/v) in 2 min, followed by isocratic elution with the latter solvent for an additional 5.5 min. Flow rate was kept constant at 300 μL/min and a 2.5 min re-equilibration time was used between runs (protocol conformed from [39]). Mass spectrometry of eluting lipids was performed using positive mode Atmospheric Pressure Chemical Ionization (APCI) in the range from 200 to 1100 Da and a resolution of 120.000 at m/z 200. Detected lipids are abbreviated as follows: CEfrag = the cholesterol fragment ion detected that originates from the indicated cholesteryl-ester; Cer = ceramide; DG = diacylglycerol; sterol = indicated free sterols; TG = triacylglycerol; TG O- = is an ether linked triglyceride (mono-alkyl, di-acyl-glycerol); TGfrag = detected diacylglycerol fragment ion originating from a triacylglycerol.

Raw LC–MS data files were converted to mzML format by msconvert from the ProteoWizard toolbox, using vendor peak picking to create centroided data. Data was analyzed using XCMS version 4.2 running under R version 4.4 and included peak-picking, grouping, alignment, and the forced integration of missed peaks [40].

### Statistical analysis

At least three biological replicates were performed for each experiment. Statistical analysis was carried out with IBM SPSS Statistics for Windows (v25; IBM Corp., Armonk, NY, United States). Data on LD parameters (i.e., size, count, clustering) consisted of numerous single-cell measurements per replicate. Three biological replicates were performed, and normality of the data distribution was evaluated at the aggregated level using the Shapiro–Wilk test. Due to the non-normal distribution of these parameters and the limited number of biological replicates, nonparametric testing was applied. The Kruskal–Wallis H test was used to compare distributions between groups. Test results are reported as the test statistic (χ^2^) with the corresponding degrees of freedom (df) indicated in parentheses, where degrees of freedom equal the number of groups minus one (e.g., χ^2^(4) for five groups). Post hoc pairwise comparisons were performed using Dunn’s (1964) procedure with Bonferroni adjustment. Outliers were observed and retained in the dataset after careful examination of the images eliminating the presence of discernible artifacts. LC–MS data were analyzed with one-way ANOVA, followed by a Tukey’s post hoc test for pairwise comparisons. Statistical significance was determined at *P* < 0.05, specifically comparing DES- or KTZ-exposed groups to their respective vehicle controls.

## Results

### Neutral lipid dye comparison

**Figure 2:**
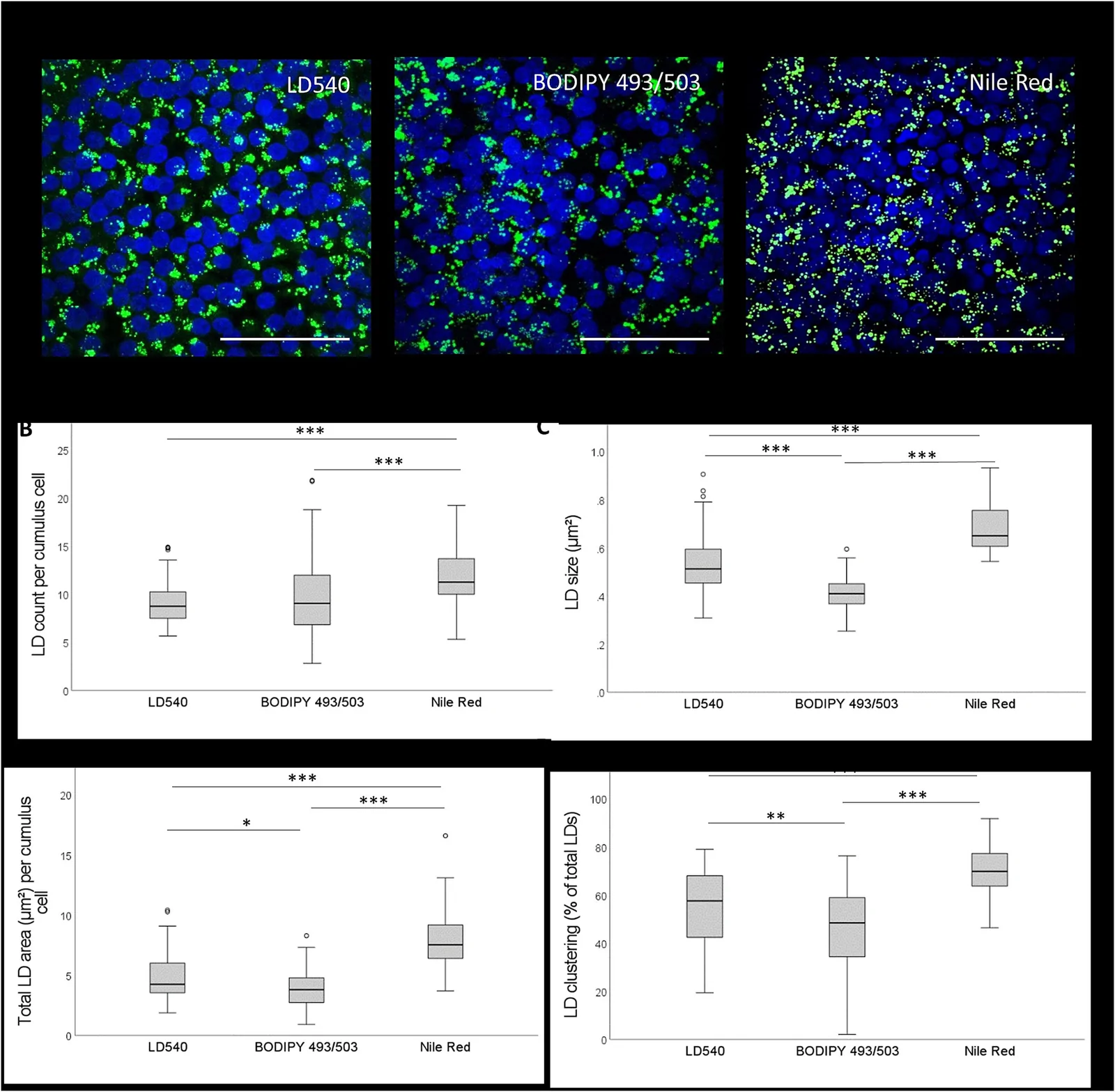
(A) Representative images of cumulus cells, from in vitro matured bovine COCs, stained with the neutral lipid dyes LD540, BODIPY 493/503, or Nile Red (LD are small ball shaped). Cumulus cell nuclei (large oval structures) were stained with Hoechst 33342. Scale bars represent 50 μm. To compare the neutral lipid dyes, images were analyzed with an automated image analysis pipeline developed in NIS Elements software (see Methods) to calculate (B) LD count, (C) LD size (μm^2^), (D) total LD area (μm^2^) per cumulus cell, and (E) LD clustering. Data represent three independent experiments, with circles indicating values more than 1.5× IQR below Q1 or above Q3. *n* = 3, r = 10 COCs for each experimental exposure. Statistical significance ^*^P < 0.05, ^**^P < 0.01, ^***^P < 0.001.

A comparison among different neutral lipid dyes was carried out to determine the most suitable dye for the visualization and characterization of lipid droplets in bovine CCs. Bovine cumulus-oocyte complexes (COCs) matured in vitro under control conditions were stained with the neutral lipid dyes LD540, BODIPY 493/503, or Nile Red (representative Figure 2A). For LD540, there was a median of 9 (IQR: 3) LDs per CC, comparable to the median of 9 (IQR: 5) LDs per CC recorded in COCs stained with BODIPY 493/503 (Figure 2B). COCs stained with Nile Red had a median of 11 (IQR: 4) LDs per CC, which was higher than the groups of LD540 (*P* < 0.001) and BODIPY 493/503 (*P* < 0.001). The average LD size was statistically significantly different for all dye groups (Kruskal–Wallis test: χ^2^(2) = 167, *P* < 0.001; Figure 2C). The smallest LD size was recorded in CCs stained with BODIPY 493/503 group (0.41 μm^2^, IQR: 0.1), followed by CCs stained with LD540 (0.51 μm^2^, IQR: 0.1) and Nile Red (0.65 μm^2^, IQR: 0.2). Analysis of the total LD area per cumulus cell also showed significant differences between the three dye groups (Kruskal–Wallis test: χ^2^(2) = 124, *P* < 0.001; Figure 2D), similar to the differences observed for LD size, with a lower total LD area for BODIPY 493/503 (3.78 μm^2^, IQR: 2.1), followed by LD540 (4.24 μm^2^, IQR: 2.5) and Nile Red (7.51 μm^2^, IQR: 2.8). Statistical analysis revealed significant differences in LD clustering among the dye groups (Kruskal–Wallis test: χ^2^(2) = 92, *P* < 0.001; Figure 2E). The highest percentage of clustering was recorded for LDs of CCs stained with Nile Red (70%, IQR: 14), followed by LD540 (58%, IQR: 26) and BODIPY 493/503 (48%, IQR: 25).

Although statistically significant differences were observed between the tested dyes, these were relatively small. Further experiments were carried out with LD540 due to practical advantages of this dye, including having better photostability and spectral resolution, as well as not requiring cell permeabilization.

### DES and KTZ effects on LDs in bovine cumulus cells

To study the effects of endocrine disrupting chemicals on lipid droplets of cumulus cells, bovine COCs were matured in vitro and exposed during the entire maturation period of 24 h to DES (10^−9^ M, 10^−7^ M, 10^−5^ M), KTZ (10^−8^ M, 10^−7^ M, 10^−6^ M), or the vehicle (DMSO) (for number of COCs and cumulus cells analyzed, see Supplementary Table S1). COCs stained with LD540 were imaged by confocal microscopy to assess the effects of DES and KTZ on LD parameters of cumulus cells, including LD count, LD size, total LD area per cell, and LD clustering (results summarized in Table 1).

**Table 1:** Summary of DES or KTZ effects on LD characteristics of cumulus cells of bovine COCs. Bovine COCs were matured in vitro and exposed to either DES or KTZ during maturation. LD parameters were investigated in CCs via confocal microscopy after LD540 staining. LD parameters include LD count per cumulus cell nucleus (*Count*), LD clustering (*Clustering*), individual LD area (*Area*), and total LD area per cumulus cell nucleus (*Total LD area*). Note that the LD population (named here as *All*) has been spatially distinguished to the *clustered* and *dispersed* subpopulations. The relevant data can be seen in Figures 3–6. Reported *P*-values correspond to Kruskal–Wallis test, assessing overall differences among exposure groups. *n* = 3, r = 10 amount of cumulus cells counted see Supplementary Table S1

	LD group	DES exposure	KTZ exposure
LD count	All	↓ 10^−5^ M, *P* < 0.001	No effect
	Clustered	↓ 10^−5^ M, *P* < 0.001	↑ all concentrations, *P* < 0.01
	Dispersed	↓ 10^−5^ M, *P* < 0.001	↓ 10^−8^ M, 10^−6^ M, *P* < 0.01
Clustering		No effect	↑ all concentrations, *P* < 0.001
LD size	All	↑ all concentrations, *P* < 0.001	↑ all concentrations, *P* < 0.001
	Clustered	↑ 10^−5^ M, *P* < 0.001	↑ all concentrations, *P* < 0.001
	Dispersed	↑ all concentrations, *P* < 0.001	↑ all concentrations, *P* < 0.001
Total LD area	All	↓ 10^−5^ M, *P* < 0.001	↑ all concentrations, *P* < 0.001

#### LD count

**Figure 3:**
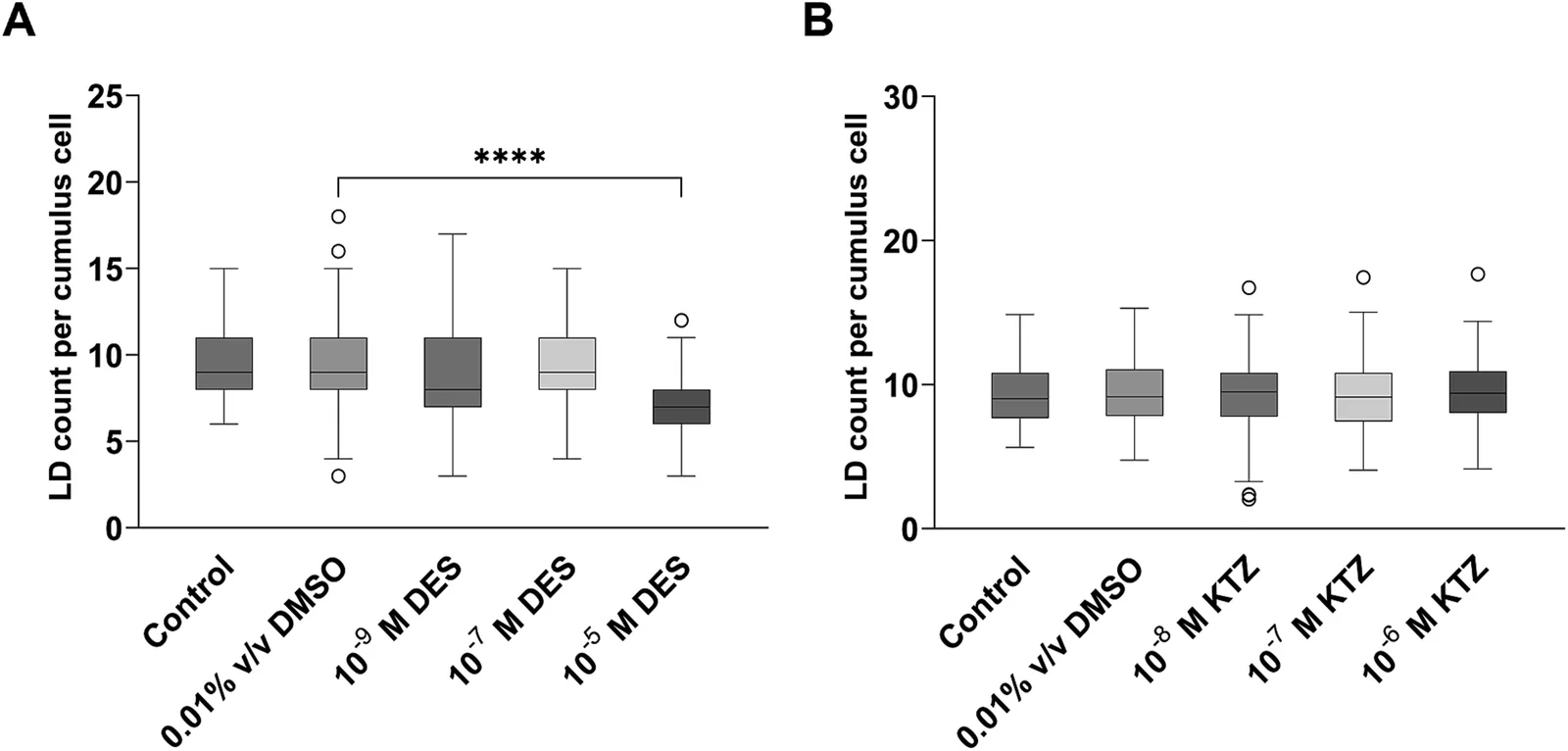
Lipid droplet count in bovine cumulus cells exposed to the EDCs (A) DES or (B) KTZ. Data represent three independent experiments with circles indicating values more than 1.5× IQR below Q1 or above Q3. *n* = 3, r = 15 COCs for each experimental exposure. Statistical significance ^***^P < 0.001.

A median of 9 (IQR: 3) lipid droplets were present per cumulus cell nucleus of the vehicle-treated groups (0.01% v/v for DES-exposure; 0.1% v/v DMSO for KTZ-exposure). Cumulus cells exposed to 10^−9^ M DES and 10^−7^ M DES had a median LD count of 8 (IQR: 4) and 9 (IQR: 3), respectively, and these values did not significantly differ from the vehicle-treated group. Exposure to the highest concentration of DES (10^−5^ M) resulted in statistically significantly lower median LD count of 7 (IQR: 2) per cumulus cell nuclei (Kruskal–Wallis test: χ^2^(4) = 123, *P* < 0.001; Figure 3A), compared to the vehicle control. KTZ exposure had no significant effect on LD count in cumulus cells at any concentration, when compared to the vehicle-treated group (Figure 3B).

#### LD size

**Figure 4:**
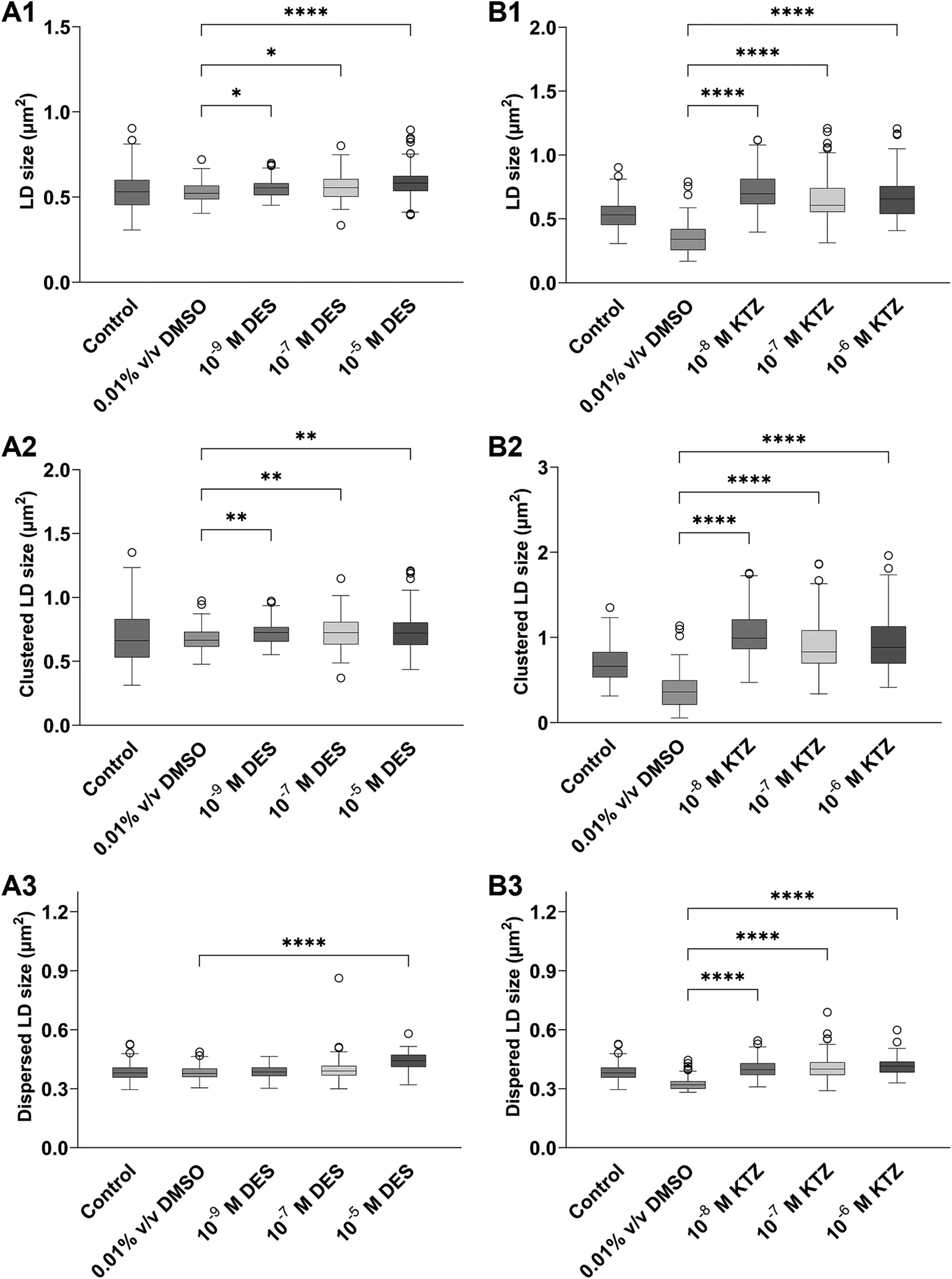
Lipid droplet size in bovine cumulus cells exposed to DES (A1–A3) or KTZ (B1–B3). Lipid droplet size of all LDs (DES: A1; KTZ: B1) was calculated. LDs were divided in two distinct subpopulations based on their spatial distribution, namely the clustered (DES: A2; KTZ: B2) and dispersed (DES: A3; KTZ: B3) LD subpopulations, that were also analyzed separately. Data represent three independent replicates with circles indicating values more than 1.5× IQR below Q1 or above Q3. Asterisks represent values more than 3.0× IQR above Q3. *n* = 3, r = 15 COCs for each experimental exposure. Statistical significance ^*^P < 0.05, ^**^P < 0.01, ^***^P < 0.001.

LD size was also investigated, and differences among groups are reported as follows. Cumulus cells of COCs exposed to DES during in vitro maturation showed an altered lipid droplet size (μm^2^) (Figure 4A1–A3). This effect was concentration-dependent and more pronounced at the highest tested DES concentration (10^−5^ M). The median LD size of cells exposed to 10^−9^ M (0.55 μm^2^, IQR: 0.1, *P* = 0.042), 10^−7^ M (0.56 μm^2^, IQR: 0.1, *P* = 0.012), or 10^−5^ M (0.58 μm^2^, IQR: 0.1, *P* < 0.001) DES was statistically significantly higher compared to the vehicle control group (0.52 μm^2^, IQR: 0.1, Kruskal–Wallis test: χ^2^(4) = 41, *P* < 0.001).

The size of dispersed and clustered LDs was also separately evaluated. Clustered LDs in cumulus cells exposed to any of the tested DES concentrations had an average size that was larger than in the vehicle control group (χ^2^(4) = 23, *P* < 0.001; Figure 4A2). When analyzing the dispersed LDs, only cumulus cells exposed to 10^−5^ M DES (0.44 μm^2^, IQR: 0.1) had a larger mean LD size compared to the vehicle-control group (0.38 μm^2^, IQR: 0.1, *P* < 0.001; Figure 4A3).

The median lipid droplet size of bovine cumulus cells exposed to KTZ concentrations of 10^−8^ M (0.7 μm^2^, IQR: 0.2), 10^−7^ M (0.61 μm^2^, IQR: 0.19), and 10^−6^ M (0.66 μm^2^, IQR: 0.18) was higher compared to the vehicle group (0.53 μm^2^; IQR: 0.13, χ^2^(4) = 182, *P* < 0.001) (Figure 4B1). A similar increase in LD size was observed for both the clustered (χ^2^(4) = 181, *P* < 0.001; Figure 4B2) and dispersed (χ^2^(4) = 77, *P* < 0.001; Figure 4B3) LDs, at all KTZ concentrations.

#### Total LD area

**Figure 5:**
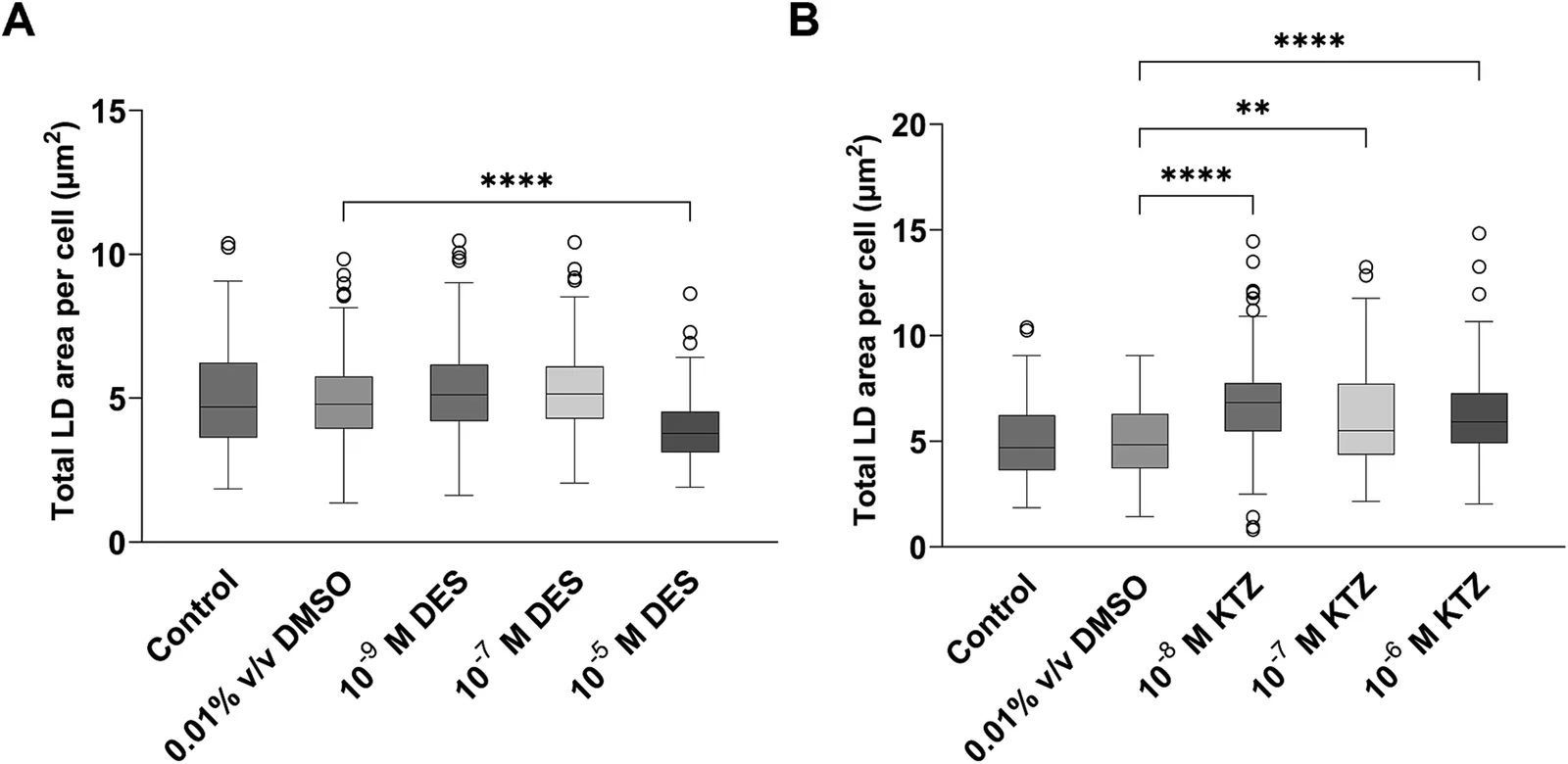
Total LD area per cumulus cell in bovine COCs exposed to (A) DES or (B) KTZ during COC in vitro maturation. Data represent three independent replicates with circles indicating values more than 1.5× IQR below Q1 or above Q3. *n* = 3, r = 15 COCs for each experimental exposure. Asterisks represent values that are more than 3× IQR above Q3. Statistical significance ^**^P < 0.01, ^***^P < 0.001.

Another parameter that could be analyzed from the adopted image analysis pipeline is the total LD area, calculated from LD size and count, per cumulus cell. A reduction in the total LD area was observed only in cumulus cells exposed to 10^−5^ M DES (3.78 μm^2^, IQR: 2.6; *P* < 0.001), compared to the vehicle control group (4.8 μm^2^, IQR: 1.8) (Kruskal–Wallis test: χ^2^(4) = 78, *P* < 0.001). This effect was not observed at other tested DES concentrations (i.e., 10^−9^ M or 10^−7^ M) (Figure 5A). The total LD area recorded in cumulus cells exposed to 10^−8^ M KTZ (6.84 μm^2^, IQR: 2.3; *P* < 0.001), 10^−7^ M KTZ (5.51 μm^2^, IQR: 3.3; *P* = 0.009), and 10^−6^ M KTZ (5.92 μm^2^, IQR: 2.4; *P* < 0.001) (Figure 5B) was higher compared to the vehicle control group (4.84 μm^2^, IQR: 2.4) (Kruskal–Wallis test: χ^2^(4) = 72, *P* < 0.001).

#### LD clustering

**Figure 6:**
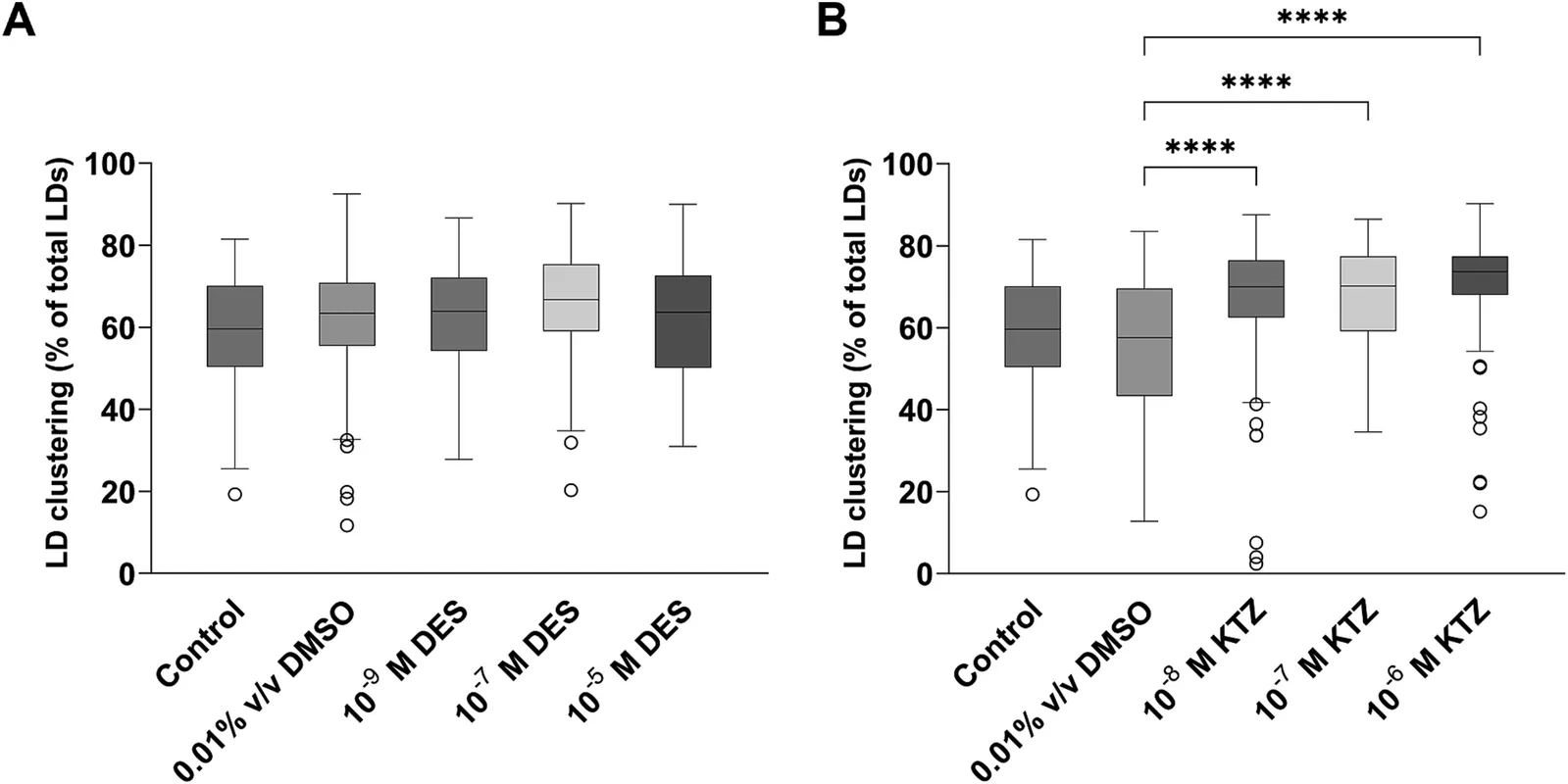
LD clustering in bovine cumulus exposed to (A) DES or (B) KTZ during COC in vitro maturation. The data represent three independent experiments with circles indicating values more than 1.5× IQR below Q1. *n* = 3, r = 15 COCs for each experimental exposure. Asterisks represent values that are more than 3.0× IQR below Q1. Statistical significance ^***^P < 0.001.

To analyze aspects of LD spatial distribution, the degree of LD clustering was evaluated by calculating the percentage of LDs in close proximity participating in clusters (see Material and Methods). Exposure of COCs to DES did not affect LD spatial distribution. LD clustering in DES-treated cumulus cells (10^−9^ M, 64%, IQR: 18; 10^−7^ M, 67%, IQR: 16; 10^−5^ M, 64%, IQR: 22) was comparable to the vehicle group (63%, IQR: 15) (Figure 6A). COCs treated with 10^−5^ M DES exhibited a reduced LD count, as shown in Figure 3A. Due to a similar reduction of cell count in both the dispersed and clustered LD subpopulations (see Supplementary Figure S1), no apparent changes were observed in the percentage of LD clustering.

Exposure of COCs to KTZ, at any concentration tested, led to statistically significant differences in LD clustering (Kruskal–Wallis test: χ^2^(4) = 118, *P* < 0.001) (Figure 6B). The median LD clustering of cumulus cells exposed to 10^−8^ M KTZ (70%, IQR: 13, *P* < 0.001), 10^−7^ M KTZ (70%, IQR:18, *P* < 0.001), and 10^−6^ M KTZ (74%, IQR: 9, *P* < 0.001) was higher compared to the vehicle control group (58%, IQR: 26). In cumulus cells exposed to 10^−8^ M or 10^−6^ M KTZ, there was a reduction of dispersed LD count concurrent with an increase of clustered LDs. In cumulus cells exposed to 10^−7^ M KTZ, an increase of clustered LDs was recorded without any changes in dispersed LD count (see Supplementary Figure S2).

### DES and KTZ effects on lipid composition of cumulus cells and oocytes

Based on the previously reported data on LD characterization by confocal imaging, it is plausible that both DES and KTZ may have an effect on neutral lipid content. To further evaluate this, and to corroborate the relevance of imaging data, we investigated the neutral lipid content in bovine cumulus cells by liquid chromatography-mass spectrometry (LC–MS). COCs were exposed to the vehicle solvent, DES, or KTZ during in vitro maturation, as previously described. Cumulus cells were separated from oocytes, and lipids of both cumulus cells and oocytes were separately extracted and further processed to be analyzed by LC–MS. In untreated cumulus cells, the levels of cholesteryl-esters (CE), diacylglycerol (DG) and triacylglycerol (TG) were approximately 143:75:210 picomoles per cumulus layer, respectively. In untreated oocytes, the respective CE:DG:TG levels were approximately 1.1:3.4:13.5 picomoles per oocyte. Note that in the extracted neutral lipid fraction of cumulus and oocytes, only TG and CE are the primary and exclusive components of LDs. While DG are co-extracted, they predominantly reside at the lipid monolayer surrounding LDs or at cellular lipid bilayer membranes. During lipid extraction, RP-HPLC elution and MS detection, free DG as well as free sterols and ceramides are also detected in both cumulus cells and oocytes.

**Figure 7:**
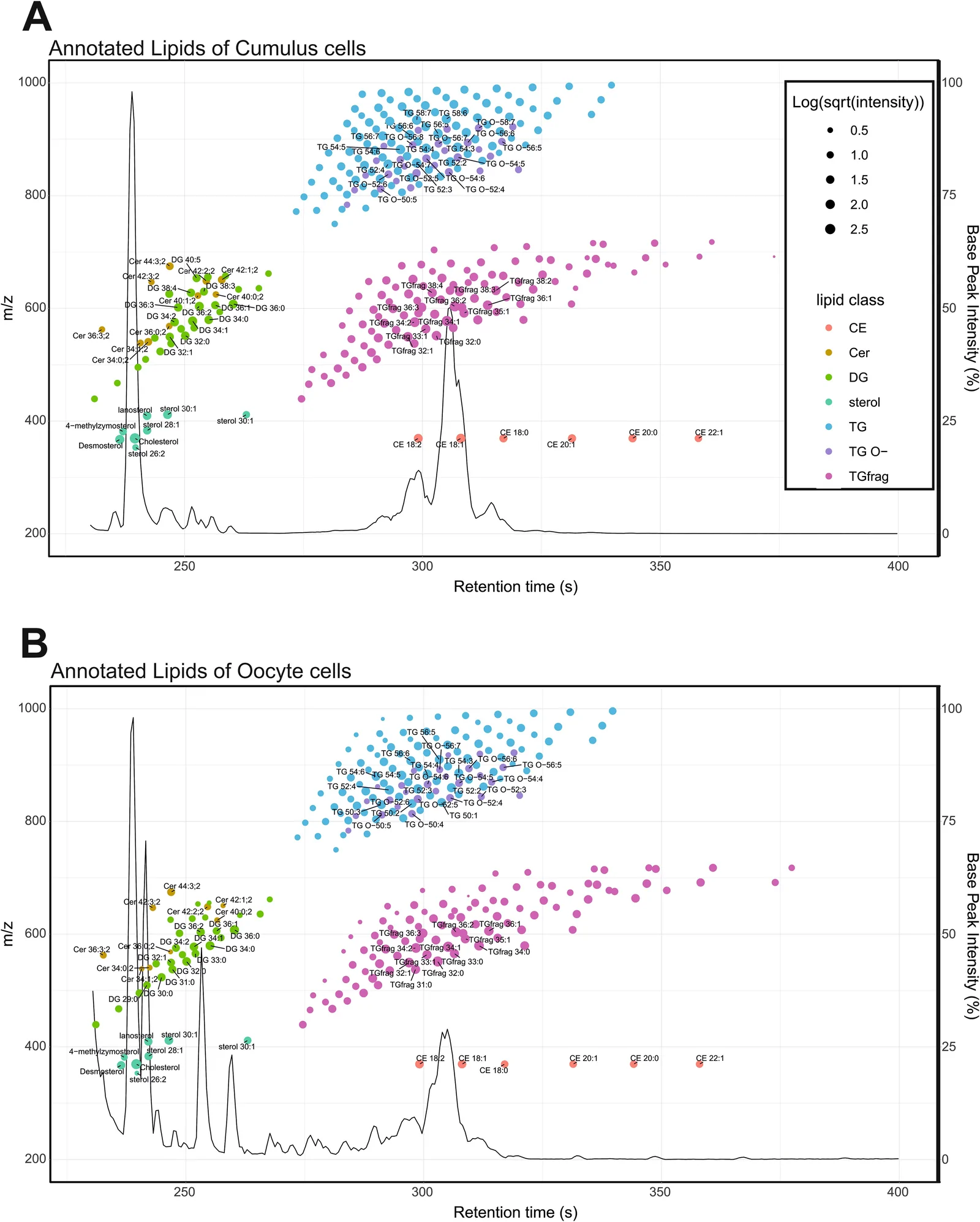
Annotation of identified extracted lipids from (A) cumulus cells and (B) oocytes after RP-HPLC elution. The *X* axis represents retention time in seconds. On the *Y* axis, the molecular mass-to-charge ratio (m/z) is depicted. Each colored dot belongs to one molecular lipid species. The diameter of each dot provides information on the relative abundance in Log(sqrt(intensity)) of each particular neutral lipid ion (see figure legend). Colors refer to specific classes of lipids (see figure legend). Abbreviations used are: CEfrag = the cholesterol fragment ion detected that originates from the indicated cholesteryl-ester; Cer = ceramide; DG = diacylglycerol; sterol = indicated free sterols; TG = triacylglycerol; TG O- = is an ether linked triglyceride (mono-alkyl, di-acyl-glycerol); TGfrag = detected diacylglycerol fragment ion originating from a triacylglycerol. Note that CEfrag and TGfrag are fragment ions, formed post-elution from the RP-HPLC and during the ionization process within the mass spectrometer.

All detected molecular species of lipids that eluted from the reverse-phase (RP) high performance liquid chromatography (HPLC) column were identified using our lipidomic platform. The full lipid composition of untreated cumulus cells and oocytes is depicted in Figure 7. The neutral lipids were detected as intact ions of TG (*TG*) or their DG fragment ions (*TGfrag*), as well as fragment ions of CE (*CEfrag*). TG, TGfrag, and CE form the majority of neutral lipids present within the lipid core of the LDs.

### Lipid class composition after KTZ or DES exposure in cumulus cells and oocytes

**Figure 8:**
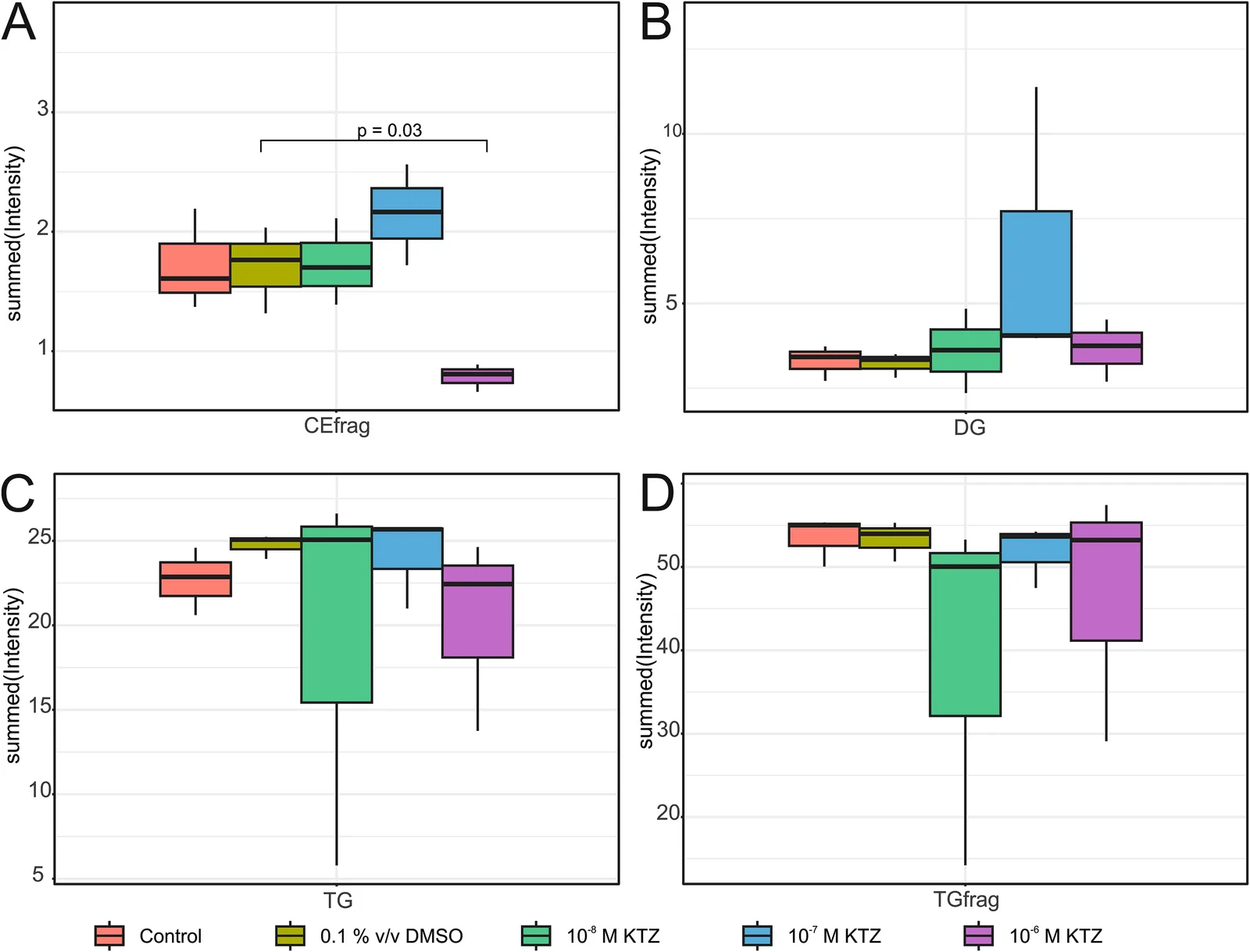
Relative intensity of lipid classes normalized to total neutral lipid, obtained from cumulus cells exposed to ketoconazole (KTZ). (A) CEfrag intensity, (B) DG intensity, (C) TG intensity, and (D) TGfrag intensity. *Y* axis represents summed intensity and *X* axis represents the incubation conditions (control, DMSO vehicle-control, 10^−8^ M KTZ, 10^−7^ M KTZ, 10^−6^ M KTZ)*. n* = 3, r = 10 cumuli for each experimental exposure.

**Figure 9:**
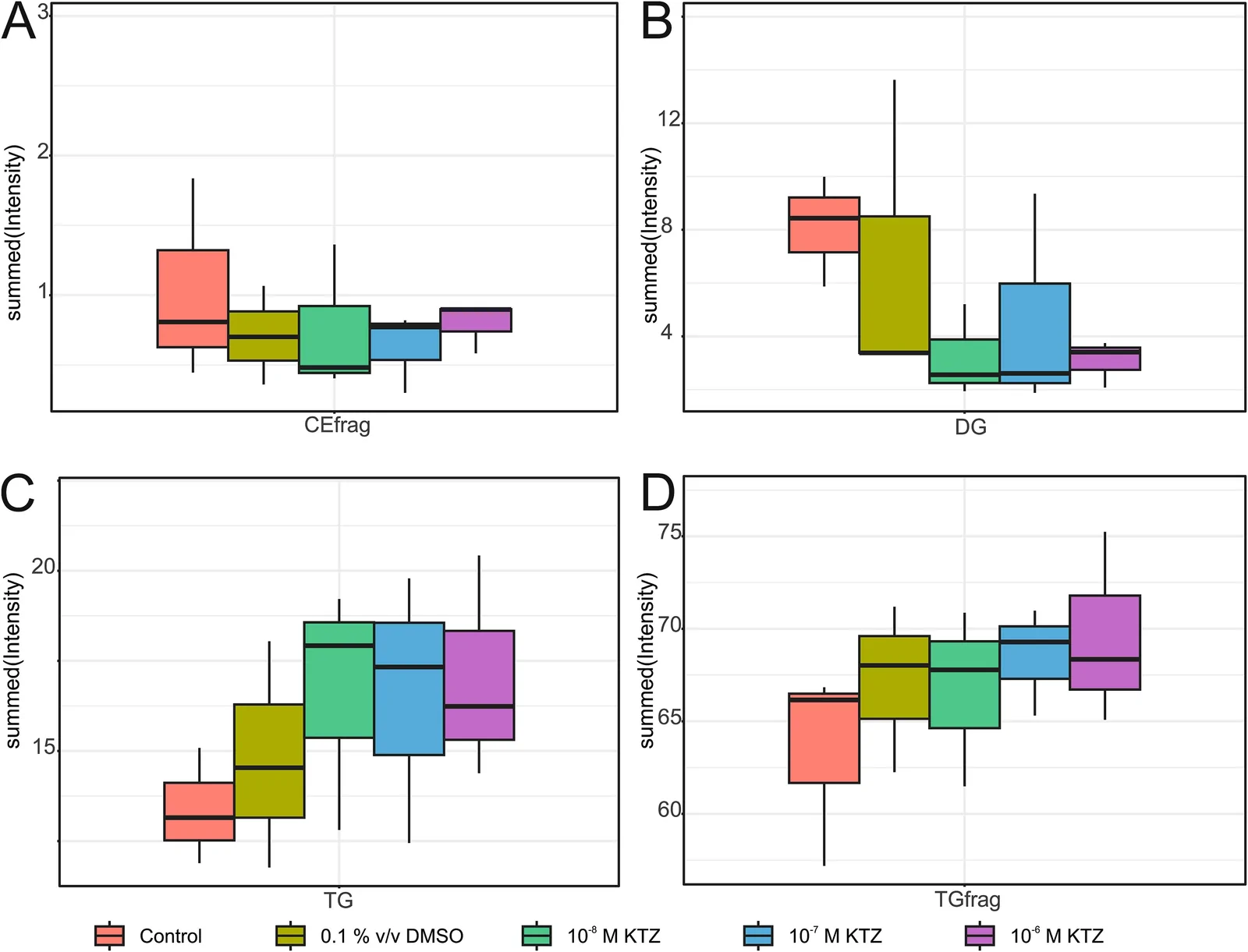
Relative intensity of lipid classes normalized to total neutral lipid, obtained from oocytes exposed to ketoconazole (KTZ). (A) CEfrag intensity, (B) DG intensity, (C) TG intensity, and (D) TGfrag intensity. *Y* axis represents summed intensity and *X* axis represents the incubation conditions (control, DMSO vehicle-control, 10^−8^ M KTZ, 10^−7^ M KTZ, 10^−6^ M KTZ). *n* = 3, r = 10 oocytes for each experimental exposure.

**Figure 10:**
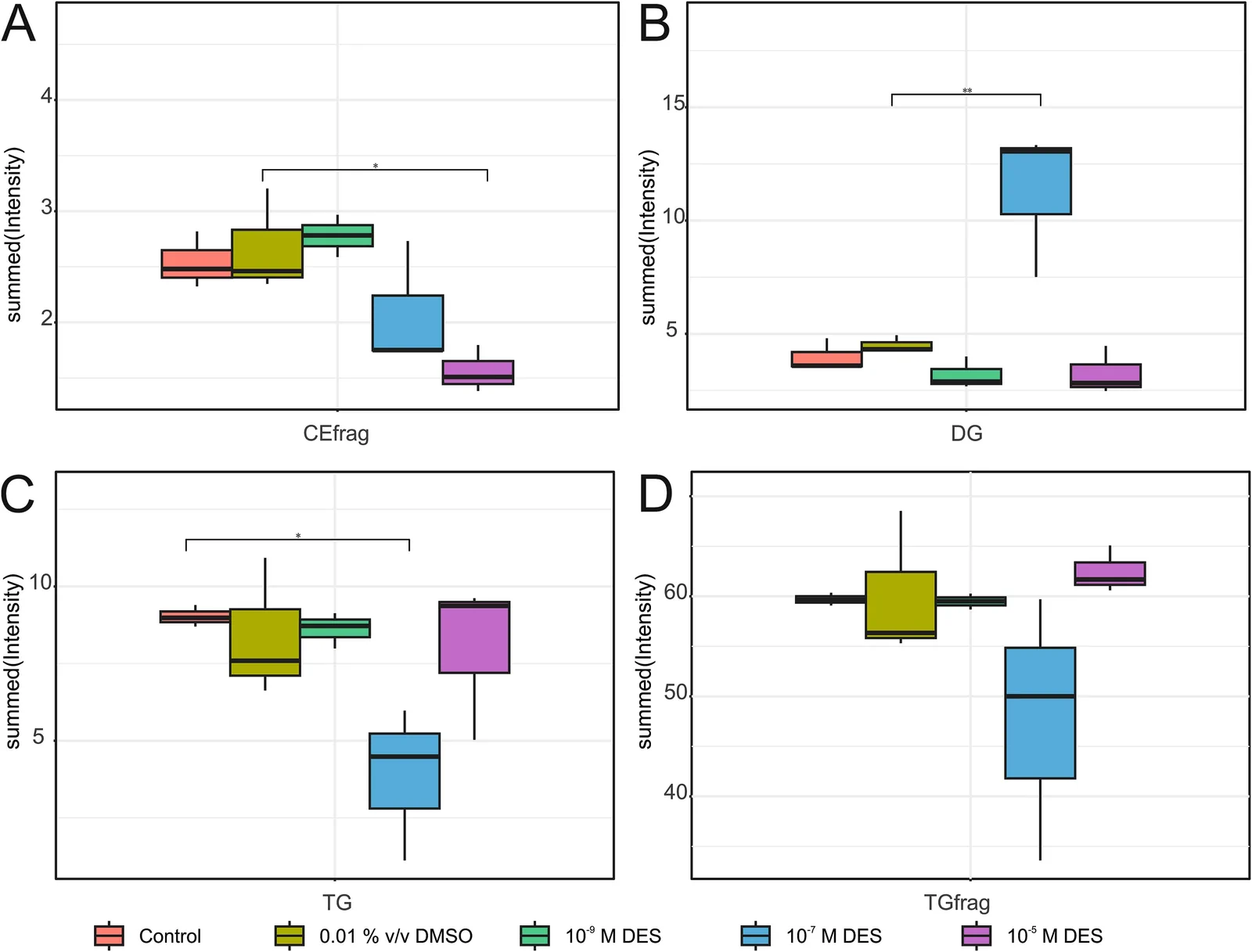
Relative intensity of lipid classes normalized to total neutral lipid, obtained from cumulus cells exposed to diethylstilbestrol (DES). (A) CEfrag intensity, (B) DG intensity, (C) TG intensity, and (D) TGfrag intensity. *Y* axis represents summed intensity and *X* axis represents the incubation conditions (control, DMSO vehicle-control, 10^−9^ M DES, 10^−7^ M DES, 10^−5^ M DES). *n* = 3, r = 10 cumuli for each experimental exposure.

**Figure 11:**
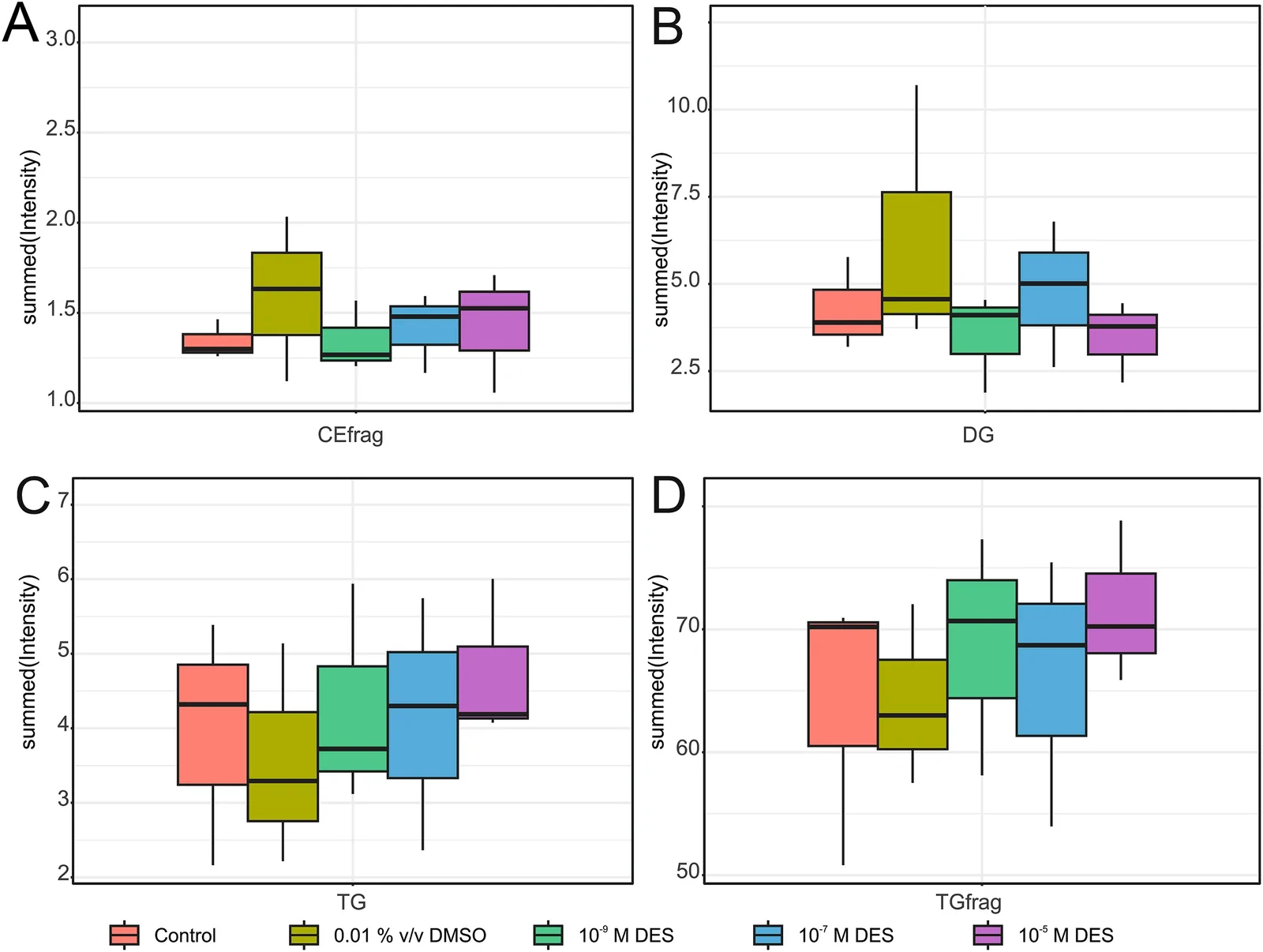
Relative intensity of lipid classes normalized to total neutral lipid, obtained from oocytes exposed to diethylstilbestrol (DES). (A) CEfrag intensity, (B) DG intensity, (C) TG intensity, and (D) TGfrag intensity. *Y* axis represents summed intensity and *X* axis represents the incubation conditions (control, DMSO vehicle-control, 10^−9^ M DES, 10^−7^ M DES, 10^−5^ M DES). *n* = 3, r = 10 oocytes for each experimental exposure.

In principle, all ions depicted in Figure 7 were present in all oocyte and cumulus extracts. The effects of KTZ and DES on the quantity and composition of lipid classes (i.e., CEfrag, TG, TGfrag, and DG ion density levels; for explanation see Figure 7) were first analyzed on the basis of their normalization to the total neutral lipid content (see Figures 8–11).

The highest tested concentration of KTZ (10^−6^ M) caused a significant and almost complete decrease of cholesteryl-esters in cumulus cells (*P* = 0.003, Figure 8A), while no changes were noted for DG, TG, and TGfrag (Figure 8B–D, respectively). In the oocyte, there were no significant changes noted in CEfrag, nor in the total amount of DG, TG, and TGfrag levels (Figure 9). A trend to detect less DG ions (Figure 9B) and more intact (TG) or fragmented TG (TGfrag) ions (Figure 9C and D) with increasing KTZ concentration, was observed, but this was not statistically significant.

A significant decrease in CEfrag was noted in cumulus cells exposed to the highest tested concentration of 10^−5^ M DES (Figure 10A). DG, TG, and TGfrag levels were comparable to that of the control and vehicle control in cumulus cells exposed to 10^−9^ M and 10^−5^ M DES (Figure 10B–D). Exposure of cumulus cells to 10^−7^ M DES resulted in significantly higher DG levels, while both the TG and TGfrag intensities were concomitantly lower (Figure 10B–D). It is noteworthy that the total amount of DG, TG, and TGfrag combined remained comparable between the 10^−7^ M DES-treated group with all other experimental groups. In oocytes, DES did not significantly alter neutral lipid class composition at all concentrations tested (Figure 11).

### Molecular species composition of each lipid class after KTZ or DES exposure in cumulus cells and oocytes

For each lipid class (CEfrag, DG, TG, and TGfrag), the lipid species composition, defined by fatty acid chain carbon atoms and double bonds present, was analyzed. The intensities were normalized to total CEfrag, total DG, total TG, and total TGfrag intensities, respectively. In Supplementary Figures S3–S10, the number of carbon double bonds and the amount of carbon atoms from participating fatty acids/alkyl chains are provided for cumulus cells and oocytes, under KTZ and DES conditions. None of the tested conditions (i.e., KTZ: 10^−8^ M, 10^−7^ M, 10^−6^ M; DES: 10^−9^ M, 10^−7^ M, 10^−5^ M) affected lipid species composition when compared to the control and vehicle-control samples.

## Discussion

Chemicals are a necessity accommodating the modern way of living, and as a result, the human population is exposed to a variety of chemicals. EDCs have the potential to cause adverse health effects, including effects on reproduction in females [4]. Evidence from experimental animal studies has shown EDC-induced adverse effects in COCs. For example, perfluorohexane sulfonate (PFHxS) has been shown to affect bovine oocyte maturation and reduce embryonic developmental competence [41]. We have previously shown that DES and KTZ affected steroidogenesis in bovine COCs, though only DES reduced bovine oocyte maturation and blastocyst formation [32, 33]. Studies have shown that DES and KTZ can affect cholesterol biosynthesis in human and rat ovarian models [42, 43]. Here, we have explored the effects of DES and KTZ on COC lipid metabolism, via analysis of LDs and neutral lipids, a majorly underexplored potential target of EDC action in the COC. LDs are highly dynamic organelles with various roles (e.g., energy storage, lipid homeostasis, and cell signaling), consisting of a phospholipid monolayer that encloses a neutral lipid core primarily composed of TGs and CEs. COCs are dependent on lipid metabolism and dysregulation of LDs may impede proper maturation of the complex and subsequent embryonic development [44].

### Neutral lipid dye selection

LD characteristics in cumulus cells were compared after staining with three commonly used neutral lipid dyes (i.e., BODIPY 493/503, Nile Red, and LD540). LD count was higher in CCs stained with Nile Red compared to CCs stained with BODIPY 493/503 and LD540, with the latter two dyes showing a comparable LD count. It has been argued that Nile Red has lower specificity compared to other neutral lipid dyes, as it may enter other vesicles with a hydrophobic core (e.g., lysosomes) or bind proteins with a hydrophobic domain [45–47]. To verify the (un)specificity of Nile Red, it would be valuable to perform co-labeling against LD-specific markers (e.g., perilipins) in the future. Due to the possibility that the discrepancy in LD count is attributable to Nile Red not being strictly specific to LDs, this dye was omitted from further experiments.

The average LD size recorded in CCs was different for all lipid dyes, with the highest size recorded for Nile Red and the lowest for BODIPY 493/503. However, the possibility that dye-specific staining protocols may have influenced size measurements cannot be excluded. For example, for both BODIPY 493/503 and Nile Red, but not LD540, a permeabilization step with the detergent Triton-X was used prior to staining. Permeabilization may lead to lipid solubilization and has formerly been associated to changes of the LD-associated proteome [48]. Permeabilization may thus have resulted in alterations of the LD structure and properties. Alternatively, the inherent properties of each dye may account for the observed LD size differences. In particular, the order of LD size reduction per dye follows the increase in dye lipophilicity (Nile Red > LD540 > BODIPY 493/503; partition coefficient logP of 5, 4.3, and 3.5, respectively) [37, 49]. Finally, Nile Red is known to bind to phospholipids of the LD membrane, as well as hydrophobic domains of membrane proteins. Although binding of Nile Red to phospholipids causes a spectral shift, differentiating it from Nile Red emission when within a highly hydrophobic environment (i.e., neutral lipid core), it may still be detected in our setup and expand the detected LD signal across the LD membrane [50].

LD540 was selected over BODIPY 493/503 for further experiments due to its comparatively better spectral resolution, photostability, and higher fluorescence intensity, as reported by the dye developer [37]. Moreover, LD540 is preferrable in handling as its incorporation into LDs did not require cell permeabilization with detergent treatment, the concentration required is 100× lower than BODIPY 493/503, and the incubation time for staining is a fourfold shorter. These advantages could be proven useful, especially in follow-up experiments of co-labeling LDs and other organelles (e.g., mitochondria) or even live-cell imaging.

### Effects of DES and KTZ on LDs and lipids

After selecting LD540 as the reporter dye to characterize LDs in bovine COCs, the effect of KTZ and DES on lipid droplets (size, count, % clustering) in CCs of in vitro matured bovine COCs was assessed. The statistical analyses of effects are summarized in Table 1. The most pronounced effect for DES exposure was a decrease in total LD area due to a significant decrease in LD count, despite the concomitant increase of the LD size, in cumulus cells exposed to 10^−5^ M DES. When cumulus cells were exposed to KTZ, an increase in total LD area and LD clustering was observed at all tested KTZ concentrations. While deviations in LD surface may appear minor, they are considerable from a volumetric point of view. For example, an LD with a surface area of 0.52 μm^2^ (diameter: 0.81 μm, volume: 0.28 μm^3^) is by 16% smaller in volume than a 0.58 μm^2^ (diameter: 0.86 μm, volume: 0.33 μm^3^) LD and by 39% than a 0.66 μm^2^ (diameter: 0.91 μm, volume: 0.40 μm^3^) LD. The effects on LD size may hint to an altered spatial LD profile, which may translate to altered dynamics and functions of cumulus cell LDs [15].

LC–MS analysis of the LDs in oocytes and cumulus cells showed that DES and KTZ exposure did not induce changes in DG and TG levels, or their respective molecular species composition. However, the highest tested concentrations of both DES (10^−5^ M) and KTZ (10^−6^ M) led to a major (>90%) depletion of CE from the neutral lipid extracts of cumulus cells, with no such effect observed on oocytes. This depletion may be the result of increased mobilization of cholesterol esters or reduced de novo synthesis of cholesterol. The possibility that CEs are depleted from LDs via the autophagy (lipophagy) pathway, involving lysosomal degradation of lipid droplets for cholesterol release, is less likely according to our tested conditions and results [26]. This is supported by specific mobilization of cholesteryl-ester only from the neutral lipids stored in LDs, while other lipid classes were nearly unaffected. In the case of lipophagy, it is highly likely that some level of TG hydrolysis would also have been detected. Intriguingly, irrespective of the underlying mechanism, both DES and KTZ had similar effects, evidenced by the specific depletion of cholesterol-esters from cumulus LDs. The herein reported data along with previous observations on DES and KTZ effects on steroidogenesis of cumulus cells are summarized below:

(i) KTZ is a potent inhibitor of the cholesterol side-chain cleavage enzyme (P450scc/CYP11A1 involved in cholesterol to pregnenolone conversion) and 10^−6^ M KTZ was shown to block pregnenolone and progesterone formation by bovine cumulus cells [33]. Here, the same concentration of 10^−6^ M KTZ is shown to deplete cholesterol esters from the LDs of cumulus cells.
(ii) DES is an estrogen receptor agonist that was shown to increase pregnenolone and progesterone levels in cumulus cells exposed to 10^−5^ M DES [32]. Specifically, exposure to 10^−5^ M DES doubles synthesis of pregnenolone and progesterone by cumulus cells and, as shown here, leads to a concomitant and nearly complete depletion of cholesterol esters from LDs.

The importance of progesterone production in bovine cumulus oocyte maturation has been demonstrated [51], and we showed in the past that DES and KTZ elicited effects on progesterone levels [32, 33]. In correlation with these previously published data from our group, the new reported data on CE depletion from LDs of cumulus cells exposed to DES may have a functional implication in providing the matrix of mitochondria in cumulus cells with precursor cholesterol molecules for steroidogenesis [27]. The mobilized cholesterol could be directly imported into the mitochondria, via LD-mitochondrial contact sites and the action of cholesterol transporters (e.g., StAR), leading to progesterone production [52]. The possible mechanism of this specific mobilization of CE from cumulus LDs is not yet clear. It is possible that LD-associated proteins on the lipid monolayer of the LD, like a specific sterol-ester hydrolase or a protein of the perilipin family, are activated, but this needs to be elucidated further [21]. In support of this, considering the action of DES as an estrogen receptor agonist, estradiol has been shown to promote sterol esterase activity, albeit in macrophages [53]. A possible explanation of CE-specific mobilization comes from evidence suggesting that neutral lipids are not randomly organized within the hydrophobic core of LDs. A recent review described a liquid crystalline phase of CEs in the periphery of LDs, under the phospholipid/cholesterol monolayer that surrounds the hydrophobic core of an LD [54]. In lipid droplets with high ratios of CE:TG, de-mixing of CE from TG, and the transition to a liquid CE crystalline phase, are favored [55–57]. Here, we have shown that cumulus cells, as opposed to oocytes, are rich in CEs, as expected in steroidogenic cells. In theory, it is possible that the relatively high enrichment of CEs in the LDs of cumulus cells (see Results; CE:DG:TG levels of 143:75:210 picomoles per cumulus layer) may cause de-mixing of CEs, acquiring a peripheral distribution in a crystalline phase. This distribution likely enhances CE availability in the LD periphery for de-esterification by membrane-associated enzymes. With this proposed mechanism, of cholesterol esterase activation in LDs with readily accessible CE in the neutral core periphery, it becomes relevant that such an effect was not observed in oocytes having low CE content (see Results; CE:DG:TG levels of 1.1:3.4:13.5 picomoles per oocyte). Alternatively, CCs may act as a barrier protecting the oocyte against DES action, but this requires further experimentation to elucidate.

We showed in the past that the two endocrine disrupting chemicals, KTZ and DES, have opposing effects on steroidogenesis [32, 33]. We showed in the current study that both KTZ and DES elicited the same effect on the depletion of cholesterol from LDs. For KTZ, the reported CE depletion could in fact be a reflection of reduced cholesterol biosynthesis, impacting the storage levels of its esterified form. In fact, KTZ has been demonstrated to act as an inhibitor of cholesterol biosynthesis [58]. However, the relative contribution in the CE content of LDs in cumulus cells of either de novo synthesized cholesterol or cholesterol obtained from lipoprotein endocytosis is not known. In in vitro cultured rat granulosa cells (precursors of cumulus cells), CE used for steroidogenesis is directly correlated to the internalized CE from lipoproteins, that is either hydrolyzed and used directly, or stored (directly as CE or after hydrolysis and re-esterification) prior to its use for steroidogenesis [59]. The relevance of these data to bovine cumulus cells, or how this may translate to the human situation, is not yet known. Alternatively, mobilization of CEs could be occurring as a result of dysregulated LD-associated proteins responsible for cholesterol release, as described for DES. An important distinction of KTZ when compared to DES is that, even though CEs may be mobilized from LDs, the released cholesterol is not expected to become used by mitochondria for progesterone production. KTZ has a well-described action in inhibiting the mitochondrial CYP450 enzymes of the steroidogenic pathway [60–62]. KTZ is known to affect multiple enzymes in the steroidogenesis pathway, e.g., CYP11A1, CYP17, and CYP19A1 [63, 64].

In this study, we used DES and KTZ as model compounds with specific endocrine modes of action to investigate potentially toxic effects. Although the tested concentrations of DES and KTZ were relatively high, pharmacological studies show that plasma levels of DES and KTZ can reach micro-molar levels, and concentrations are consistent with concentrations employed in previous screening assays [32, 33, 65, 66]. Fungicide treatments with ketoconazole are reported to be 160 mg/kg/day [66] corresponding to approx. 3^*^10^−4^ M KTZ (calculation based on the assumption that this dose is completely and evenly uptaken in the body and not degraded in one day time; thus, the actual levels are lower). However, this calculated level is 300 times higher than the highest dose KTZ used in our study. DES treatments are now abandoned in human medicine but higher dose treatments in women and animals have been used until the 1980s for the prevention of miscarriage with clear adverse effects on especially offspring [65]. In the present study, an interesting observation was the concomitant detection of TG and TG frag ion decrease and DG ion increase, specifically in cumulus cells exposed to 10^−7^ M KTZ. This observation hints to a level of TG lipolysis specific to this tested KTZ concentration, and not the lower (10^−8^ M) or higher (10^−6^ M) ones, suggesting a non-monotonic effect. Non-monotonic dose–response curves are not uncommon in endocrine toxicology and may have several biological explanations [67, 68]. Further studies should be performed with a broader test concentration range, including chemicals with other modes of action, to delineate the potential effects of EDCs on lipid metabolism in LDs, particularly in COCs, and subsequent consequences for female reproductive health. One hypothesis might be that the tested compounds may affect mitochondrial distribution in the cumulus cells in such a way that they may form contact sites with LDs and that the consequent sequestering of liberated cholesterol is similar but the mitochondrial machinery how to deal with the incoming cholesterol is different under DES and KTZ exposure. Alternative explanations such as autophagy-related events cannot be excluded, although we expect in the case of autophagy a more general depletion of other neutral lipids classes instead of the observed exclusive depletion of cholesteryl-esters. The underlying mechanisms and the functional consequences of cholesteryl-ester depletion from lipid droplets in cumulus cells involved indeed should be a matter of future research.

## Conclusion

We have utilized the bovine model of in vitro COC maturation to explore the effect of KTZ and DES, two well-known EDCs with distinct mechanisms of action, on LD size and distribution, along with neutral lipid profiling. The use of this model in exploring EDC-induced reproductive toxicity is supported by the similarities of human and bovine COC maturation, as well as the adherence to the 3R principle (i.e., to replace, reduce, and refine) in experimental animal usage. An automated fluorescence image analysis pipeline for LD characterization was developed, allowing for quantitative large-scale LD analysis. DES and KTZ, both affected LDs and neutral lipids in bovine COCs. Although most observations on the size and distribution of LDs within cumulus cells appeared minor, they may be considerable from a volumetric point of view. Moreover, both DES and KTZ led to CE depletion at the highest concentrations tested. We have previously shown a potential functional implication of CE depletion, linked to altered steroidogenesis. Further functional implications of the observed effects should be elucidated in the future to discern their potential impact on COC health.

## Consent for publication

Not applicable.

**Competing interests:** The authors declare that they have no competing interests.

## Supplementary Material

Supplementary_material_ioag119

## Data Availability

All data pobtained and calculted from LC-MS/MS (lipidomics) and Confocal Microscopy (imaging) are available in a Yoda repsoitory DOI: https://doi.org/10.24416/UU01-ETLQ1J
