# Supplementary material for: The impact of endocrine disrupting chemicals diethylstilbestrol and ketoconazole on lipid storage in in vitro matured bovine cumulus-oocyte complexes
Source: Biol Reprod. 2026 Jun 5;115(3):968–85. doi: 10.1093/biolre/ioag119 (PMC13578495; doi:10.1093/biolre/ioag119)
Supplement: Supplementary file 1 [file supplementary_material_ioag119.zip › Suppl file Fig S10 new.docx]

**Figure S10**. Fatty acid chain length of (A) cholesterol (CEfrag), (B) diacylglycerol (DG) and (C,D) triacylglycerol (TGfrag and TG), in oocytes exposed to DES. n=3, r=10 oocytes for each experimental exposure.
