# Supplementary material for: The impact of endocrine disrupting chemicals diethylstilbestrol and ketoconazole on lipid storage in in vitro matured bovine cumulus-oocyte complexes
Source: Biol Reprod. 2026 Jun 5;115(3):968–85. doi: 10.1093/biolre/ioag119 (PMC13578495; doi:10.1093/biolre/ioag119)
Supplement: Supplementary file 1 [file supplementary_material_ioag119.zip › Suppl Table 1 new.docx]

**Supplementary Table S1**: Amount of COCs and their respective cumulus cells analyzed for neutral lipid dye comparison or LD characteristics analysis with LD540 after DES or KTZ exposure.

| Neutral lipid dye comparison | | |
| --- | --- | --- |
|  | COCs | CCs |
| LD540 | 30 in groups of 10 (n=3) | 10793 |
| Nile Red | 30 in groups of 10 (n=3) | 14091 |
| BODIPY 493/503 | 30 in groups of 10 (n=3) | 13389 |
| DES/KTZ exposure | | |
| Control | 45 in groups of 15 (n=3) | 15755 |
| 0.01% v/v DMSO | 45 in groups of 15 (n=3) | 18061 |
| 10¯⁵ M DES | 45 in groups of 15 (n=3) | 20770 |
| 10¯⁷ M DES | 45 in groups of 15 (n=3) | 18943 |
| 10¯⁹ M DES | 45 in groups of 15 (n=3) | 19186 |
| 0.1% v/v DMSO | 45 in groups of 15 (n=3) | 15520 |
| 10¯⁶ M KTZ | 45 in groups of 15 (n=3) | 17991 |
| 10¯⁷ M KTZ | 45 in groups of 15 (n=3) | 19235 |
| 10¯⁹ M KTZ | 45 in groups of 15 (n=3) | 30425 |
