# Supplementary material for: The impact of endocrine disrupting chemicals diethylstilbestrol and ketoconazole on lipid storage in in vitro matured bovine cumulus-oocyte complexes
Source: Biol Reprod. 2026 Jun 5;115(3):968–85. doi: 10.1093/biolre/ioag119 (PMC13578495; doi:10.1093/biolre/ioag119)
Supplement: Supplementary file 1 [file supplementary_material_ioag119.zip › Suppl file Fig S1 new.docx]

**Supplementary Figure S1**. Count of (A) clustered LDs and (B) dispersed LDs, in bovine cumulus cells exposed DES. n=3, r=15 COCs for each experimental exposure. Circles represent values that are more than 1.5 x IQR below Q1 or above Q3. Statistical significance * p < 0.05, ** p < 0.01, *** p < 0.001

*

**

***

***
